# Augmented Physics-Based Models for High-Order Markov Filtering

**DOI:** 10.3390/s24186132

**Published:** 2024-09-23

**Authors:** Shuo Tang, Tales Imbiriba, Jindřich Duník, Ondřej Straka, Pau Closas

**Affiliations:** 1 Electrical and Computer Engineering Department, Northeastern University, Boston, MA 02115, USAclosas@ece.neu.edu (P.C.); 2 Department of Cybernetics, University of West Bohemia, Univerzitní 8, 30100 Plzeň, Czech Republic

**Keywords:** nonlinear filtering, high-order Markov, hybrid neural network

## Abstract

Hybrid physics-based data-driven models, namely, augmented physics-based models (APBMs), are capable of learning complex state dynamics while maintaining some level of model interpretability that can be controlled through appropriate regularizations of the data-driven component. In this article, we extend the APBM formulation for high-order Markov models, where the state space is further augmented with past states (AG-APBM). Typically, state augmentation is a powerful method for state estimation for a high-order Markov model, but it requires the exact knowledge of the system dynamics. The proposed approach, however, does not require full knowledge of dynamics, especially the Markovity order. To mitigate the extra computational burden of such augmentation we propose an approximated-state APBM (AP-APBM) implementation leveraging summaries from past time steps. We demonstrate the performance of AG- and AP-APBMs in an autoregressive model and a target-tracking scenario based on the trajectory of a controlled aircraft with delay-feedback control. The experiments showed that both proposed strategies outperformed the standard APBM approach in terms of estimation error and that the AP-APBM only degraded slightly when compared to AG-APBM. For example, the autoregressive (AR) model simulation in our settings showed that AG-APBM and AP-APBM reduced the estimate error by 31.1% and 26.7%. The time cost and memory usage were reduced by 37.5% and 20% by AP-APBM compared to AG-APBM.

## 1. Introduction

State estimation and filtering with noisy measurements is an essential component in numerous information processing engineering applications [1]. The filtering process usually involves two steps: the prediction through the transition model and a correction based on the observed measurements and associated model. Based on the complexity of the models, the filters can be classified into two categories: (1) linear filters, e.g., Kalman filter (KF) for the linear models [2], and (2) nonlinear filters, e.g., extended Kalman filters (EKF) [3], sigma-point Kalman filters [4,5], and particle filters [6]. $H_{\infty }$ filter is an alternative filtering technique other than Kalman filters, which works especially for unknown noise statistics and the worst-case estimation error [7]. $H_{\infty }$ filters are also applied to varies applications, e.g., network systems [8], target tracking [9], battery charging [10], etc. In this article, we focus on Kalman filter techniques with given noise statistics.

When considering nonlinear dynamics, machine learning (ML) strategies are appealing when dealing with complex models due to their flexibility and effectiveness in constructing mappings and capturing intricate patterns. However, purely data-driven ML solutions, which do not leverage the knowledge brought by physics-based models (PBM), lack interpretability of the physical meaning of estimated quantities, which is especially relevant when one aims at recovering latent states [11,12]. To incorporate the information from physics knowledge, hybrid ML algorithms are usually used to provide corrections to the estimation [13,14] through neural networks (NNs) or estimate the state directly [15]. In parallel, it has been shown that the NN parameter learning process can be interpreted as a state estimation filtering problem [16,17], which can be leveraged to design sequential training schemes that jointly estimate the state process and NN parameters. This training approach is exploited by augmented physics-based models (APBMs) [18] where physics-based models are augmented by a data-driven component that complements or learns the behaviors that physics-based models cannot represent. A survey of augmented physics-based models for navigation systems is presented in [19].

High-order Markov chains are widely applied and are able to achieve better performance in many time-series data processing applications, especially when data latency or communication delay occurs, e.g., biological sequence analysis [20], speech recognition [21], classification and detection problem [22], and autoregressive process estimation [23,24]. Current solutions to this problem usually involve a more complex system model. In those situations the APBM has great potential, where the complexity that can be captured by the NN part while the physics-based component of the model is kept simple.

In this paper, we focus on adapting the APBM approach to high-order Markov models when the system dynamics and the order of the Markovity are not given. More precisely, we augment the state of the high-order Markov model to fit the APBM and train the parameters of the NN with longer-memory data. An approximation-based method is also put forward to reduce the computational cost brought by the higher-dimensional augmented state. Section 2 introduces these two proposed APBM-based methods. Finally, a tracking experiment based on a delayed-feedback control application is discussed in Section 3 to validate the two proposed approaches. The remainder of this section quickly reviews APBMs and how high-order Markovianity is typically handled in filtering contexts.

### 1.1. Augmented Physics-Based Model

Consider the Markovian dynamics and measurement models

(1) $$\begin{array}{cc} x_{k} & =f\left(x_{k-1}\right)+w_{k-1}^{x}\\ y_{k} & =h\left(x_{k}\right)+v_{k}^{y}, \end{array}$$

where $x_{k}\in R^{d_{x}\times 1}$ is the state vector at time *k* and $y_{k}\in R^{d_{y}\times 1}$ is the measurement vector at time *k*. f(·) and h(·) are, possibly nonlinear, vector functions describing the state dynamics and measurement equations, respectively. $w_{k-1}^{x}∼N\left(0,Q^{x}\right)$ is the zero-mean Gaussian noise of dynamics model and $v_{k}^{y}∼N\left(0,R^{y}\right)$ is the zero-mean Gaussian noise of measurement model. The proposed hybrid NN framework is able to learn the dynamics (1) from both sampling data and physics knowledge. The state dynamics, as described by an APBM, can be expressed as

(2) $$\begin{array}{c} x_{k}=\overset{˘}{g}\left(\bar{f}\left(x_{k-1}\right),x_{k-1};\theta \right)+w_{k-1}^{x}, \end{array}$$

where $\bar{f}\left(\cdot \right):R^{d_{x}}\mapsto R^{d_{x}}$ is the PBM, which can be obtained from the simplification, approximation, or partial knowledge of the true dynamics model f(·). $\overset{˘}{g}\left(\cdot \right):R^{d_{x}}\times R^{d_{x}}\mapsto R^{d_{x}}$ is a vector-valued function, including a NN parameterized by $\theta \in R^{d_{\theta }}$ to compensate the mismatch of the PBM $\bar{f}$ compared with true dynamics function *f*.

Furthermore, the regularization method involving a parameter value $\bar{\theta }$ is introduced in the APBM framework [18] to prevent the NN augmentation from completely taking over the model dynamics and neglecting the physics. The value $\bar{\theta }\in R^{d_{\theta }}$ is defined such that the augmented model is equivalent to the PBM when $\theta =\bar{\theta }$: $\overset{˘}{g}\left(\bar{f}\left(x_{k-1}\right),x_{k-1};\theta =\bar{\theta }\right)=\bar{f}\left(x_{k-1}\right)$.

The learning process infers the state $x_{k}$ and the parameter vector θ estimates. Considering the Bayesian estimation training approach [16,17], based on augmentation of the state $x_{k}$ by the parameter vector $\theta_{k}$, the state-space dynamics and measurement model can be rewritten as

(3) $$\begin{array}{cc} \left[\begin{array}{c} \theta_{k}\\ x_{k} \end{array}\right]\; & =\left[\begin{array}{cc} \; & \theta_{k-1}\\ \; & \overset{˘}{g}\left(\bar{f}\left(x_{k-1}\right),x_{k-1};\theta_{k-1}\right) \end{array}\right]+\left[\begin{array}{c} w_{k-1}^{\theta }\\ w_{k-1}^{x} \end{array}\right]\\ \left[\begin{array}{c} y_{k}\\ \bar{\theta } \end{array}\right] & =\left[\begin{array}{c} h(x_{k})\\ \theta_{k} \end{array}\right]+\left[\begin{array}{c} v_{k}^{y}\\ v_{k}^{\theta } \end{array}\right], \end{array}$$

where $w_{k}^{\theta }∼N\left(0,Q^{\theta }\right)$ denotes the zero-mean Gaussian noise for NN parameter dynamics. The equation $\bar{\theta }=\theta_{k}+v_{k}^{\theta }$ in (3) serves for regularization purposes and can be perceived as a soft constraint of the APBM with respect to the PBM. It has a form of the pseudo-measurement with noise $v_{k}^{\theta }∼N\left(0,\frac{1}{\lambda }I\right)$, where λ is the user-defined parameter that controls penalization of distance between $\theta_{k}$ and $\bar{\theta }$.

### 1.2. Augmented State for High-Order Markov Models

The transition model shown in (1) actually follows a first-order Markov chain, which means the current state is independent of all previous states except the most recent one [25] (Ch. 13). In a probabilistic framework, the joint distribution for a sequence of *K* states $\left[x_{1},\cdots ,x_{K}\right]$ under such a model (ignoring the measurements) is given by

(4) $$\begin{array}{c} p\left(x_{1},\cdots ,x_{K}\right)=p\left(x_{1}\right)\prod_{k=2}^{K}p\left(x_{k}|x_{k-1}\right)\;. \end{array}$$

The first-order Markov model is a general and useful assumption in many applications, but it is restrictive in others. In reality, the sequential observations usually indicate a trend, which means the past data could provide nontrivial information to the next prediction. In this case, we model a high-order Markov chain [25] (Ch. 13) and [26], which allows the prediction to depend on a sequence of previous states. For instance, the joint distribution of the states in a second-order Markov chain is given by

(5) $$\begin{array}{c} p\left(x_{1},\cdots ,x_{K}\right)=p\left(x_{1}\right)p\left(x_{2}|x_{1}\right)\prod_{k=3}^{K}p\left(x_{k}|x_{k-1},x_{k-2}\right)\;. \end{array}$$

A common approach to deal with high-order Markov is to augment the state vector with the previous states [23,27,28] so that the model becomes first-order Markovian. The augmented-state example of a second-order linear Markov model is shown in [29] (Ch. 6). Considering a general *p*th-order Markov model for the dynamics, Equation (1) can be expressed as

(6) $$\begin{array}{cc} x_{k} & =f\left(x_{k-1},\ldots ,x_{k-p}\right)+w_{k-1}^{x}\\ y_{k} & =h\left(x_{k}\right)+v_{k}^{y}\;, \end{array}$$

where $x_{k}\in R^{d_{x}\times 1}$ is the state vector at time *k*, $y_{k}\in R^{d_{y}\times 1}$ is the measurement vector at the time *k*. To filter this model, the conventional technique is to augment the state as ${\tilde{x}}_{k}={\left[x_{k}^{⊤},\ldots ,x_{k-p+1}^{⊤}\right]}^{⊤}$ and the transition model becomes

(7) $$\begin{array}{cc} {\tilde{x}}_{k} & =\left[\begin{array}{c} f({\tilde{x}}_{k-1})\\ \left[\begin{array}{c} I_{d_{x}p\times d_{x}p}\;0_{d_{x}p\times 1} \end{array}\right]{\tilde{x}}_{k-1} \end{array}\right]+\left[\begin{array}{c} w_{k-1}^{x}\\ 0_{d_{x}p\times 1} \end{array}\right]\;, \end{array}$$

where I and 0 denote the identity and zero matrices with the dimensions indicated in the sub-indices. This augmented state (AGS) transforms the high-order Markovity into a first-order Markov model. After the augmentation, standard Bayesian filtering techniques can be used on the transformed first-order Markov model. In this article, due to the nonlinear nature of the APBM and measurement models, we employ a Cubature Kalman filter (CKF) in the experiments, which propagates the cubature points through the transition and measurement model [5], although other filtering solutions could be considered without loss of generality.

### 1.3. Contributions

State estimation in high-order Markov models presents a significant challenge due to the dependence of system dynamics on previous states. A widely-used method to address this issue is the aforementioned AGS approach. However, AGS is not applicable when (1) the full knowledge of the complex system dynamics (associated with the high-order Markov model) is unavailable or (2) the order of Markovity is unknown. In this paper, we introduce the augmented-state APBM, which leverages the APBM technique to learn system dynamics via the AGS method, allowing for state estimation even with partially known system dynamics or an unknown Markovian order. To reduce the computational burden brought by the state augmentation (based on the order of Markovity), we proposed the approximated-state APBM by using the point estimate of the posterior distribution of the previous states as the training input, while this approximation introduces a slight degradation in estimation accuracy, it significantly reduces computational costs by lowering the dimension of the state space.

## 2. Augmented-State APBM for High-Order Markov Models

In this section, we extend APBMs to cope with high-order Markov models, aiming at learning the dynamics from data while constraining the augmented model around the PBM. Since high-order Markov models considered in this paper do not lead to changes in the measurement model (1), we focus next on the transition model and associated prediction process.

### 2.1. Augmented-State APBM

APBMs are appealing when the nonlinear dynamics f(·) of the *p*th-order Markov model in (6) are not accurately known, either due to their parametric representation and/or the knowledge of the Markov process order. In this case, we employ APBM to learn the transition model

(8) $$\begin{array}{c} x_{k}=g\left(\bar{f}\left(x_{k-1}\right),x_{k-1},\ldots ,x_{k-l};\theta \right)+w_{k-1}, \end{array}$$

where $g\left(\cdot \right):\underset{l}{\underset{︸}{R^{d_{x}}\times \ldots \times R^{d_{x}}}}\mapsto R^{d_{x}}$ includes the PBM and a NN parameterized by $\theta \in R^{d_{\theta }}$. We use *g* instead of $\overset{˘}{g}$ to denote the APBM function that accounts for longer state memory. *l* is a design parameter that is intuitively assumed to be chosen such that l≥p, although simulations show that reasonable results can be obtained otherwise.

For the *p*th-order Markov chain, the predictive distribution of the state can be computed as

(9) $$\begin{array}{cc} \; & p\left(x_{k}|y_{1:k-1}\right)=\int \cdots \int p\left(x_{k}|x_{k-1},\ldots ,x_{k-p},y_{1:k-1}\right)\\ \; & \;\cdot p\left(x_{k-1:k-p}|y_{1:k-1}\right)dx_{k-1}dx_{k-2}\ldots dx_{k-p}\;. \end{array}$$

However, the joint posterior distribution $p(x_{k-1:k-p}|y_{1:k-1})$ is usually not accessible. As mentioned before, the typical approach to tackling this problem is through state augmentation. Considering a *p*th-order Markovian model (6) and the APBM (8), augmentation of the state vector with the l−1 previous states results in ${\tilde{x}}_{k}={\left[x_{k}^{⊤},\ldots ,x_{k-l+1}^{⊤}\right]}^{⊤}$. The augmented-state APBM (AG-APBM) is defined by

(10) $$\begin{array}{cc} {\tilde{x}}_{k} & =\left[\begin{array}{c} g\left(\bar{f}\left(x_{k-1}\right),x_{k-1},\ldots ,x_{k-l};\theta_{k-1}\right)\\ \left[\begin{array}{c} I_{d_{x}l\times d_{x}l}\;0_{d_{x}l\times 1} \end{array}\right]{\tilde{x}}_{k-1} \end{array}\right]+\left[\begin{array}{c} w_{k-1}^{x}\\ 0_{d_{x}l\times 1} \end{array}\right]\\ \; & \equiv \tilde{g}\left({\tilde{x}}_{k-1};\theta_{k-1}\right)+{\tilde{w}}_{k-1}^{x}\\ \theta_{k} & =\theta_{k-1}+w_{k-1}^{\theta }, \end{array}$$

where ${\tilde{w}}_{k}^{x}∼N\left(0,\mathrm{diag}[Q^{x},0_{\left(p-1)\times (p-1\right)}]\right)$ denotes the augmented state processing noise. The first row in ${\tilde{x}}_{k}$ is the original *p*th-order Markov transition model and the other rows represent the dynamics of the augmentation. For the joint state-parameter estimation we assume the same augmented measurement model as in (3) since it regularizes the NN contribution to the dynamics, i.e., $\tilde{g}\left({\tilde{x}}_{k-1};\theta =\bar{\theta }\right)=\bar{f}\left(x_{k-1}\right)$ [18]. For this augmented state, the predictive distribution is given by

(11) $$\begin{array}{cc} \; & p\left({\tilde{x}}_{k}|y_{1:k-1}\right)=\int p\left({\tilde{x}}_{k}|{\tilde{x}}_{k-1},y_{1:k-1}\right)\\ \; & \;\;\;\;\cdot p\left({\tilde{x}}_{k-1}|y_{1:k-1}\right)d{\tilde{x}}_{k-1}. \end{array}$$

The above Equation (11) has the same quantities with Equation (9), but different meaning and feasibility. The posterior $p(x_{k-1:k-p}|y_{1:k-1})$ in Equation (9) is not easy to compute because it is in the joint form. However, notice that by augmenting the vector, the posterior $p({\tilde{x}}_{k-1}|y_{1:k-1})$ is not the joint distribution anymore, but a high-dimensional simpler posterior distribution, as it only involves a single variable ${\tilde{x}}_{k-1}$. This posterior distribution is accessible during the filtering process. For example, in a Kalman filter, we recursively compute the posterior for state estimation at each time step.

### 2.2. Approximated-State APBM

The augmentation approach discussed above can lead to very high-dimensional state spaces if *l* is too large; therefore, it increases the computational cost of such a solution. This cost is mostly due to the need to solve multi-dimensional integrals in the Bayesian filtering Equation (11). To circumvent this issue, using the point estimate from the previous steps—rather than using the entire distribution—reduces the computational complexity in evaluating the aforementioned integrals. This leads to the approximated-state APBM (AP-APBM):

(12) $$\begin{array}{cc} x_{k} & =g\left(\bar{f}\left(x_{k-1}\right),x_{k-1},{\hat{x}}_{k-2},\ldots ,{\hat{x}}_{k-l};\theta_{k-1}\right)+w_{k-1}\\ \theta_{k} & =\theta_{k-1}+w_{k-1}^{\theta }, \end{array}$$

where ${\hat{x}}_{k-j}=E\left\{x_{k-j}|y_{1:k-j}\right\}$, j=2,…,l. It is worth noting that approximations have to be made to obtain the above model. Equation (13) provides a mathematical explanation of this intuition by approximating the posterior distribution previous to its use in the integral.

(13) $$\begin{array}{cc} p(x_{k-2:k-l}|y_{1:k-1}) & \approx \prod_{i=2}^{l}p\left(x_{k-i}|y_{1:k-1}\right)\approx \prod_{i=2}^{l}p\left(x_{k-i}|y_{1:k-i}\right)\\ \; & \approx \prod_{i=2}^{l}\delta \left(x_{k-i}-E\left\{x_{k-i}|y_{1:k-i}\right\}\right), \end{array}$$

where δ(·) is the Dirac delta function. Note that in the above approximation, we first assume that the posterior distributions at each time step are independent, and then we neglect the dependency between states and future measurements. In addition, we finally replace the posterior distribution of the states with their point estimates, which is equivalent to the model in (12).

Finally, we present the AP-APBM

(14) $$\begin{array}{cc} \left[\begin{array}{c} \theta_{k}\\ x_{k} \end{array}\right]\; & =\left[\begin{array}{cc} \; & \theta_{k-1}\\ \; & g\left(\bar{f}\left(x_{k-1}\right),x_{k-1},{\hat{x}}_{k-l:k-2};\theta_{k-1}\right) \end{array}\right]+\left[\begin{array}{c} w_{k-1}^{\theta }\\ w_{k-1}^{x} \end{array}\right]\\ \left[\begin{array}{c} y_{k}\\ \bar{\theta } \end{array}\right] & =\left[\begin{array}{c} h(x_{k})\\ \theta_{k} \end{array}\right]+\left[\begin{array}{c} v_{k}^{y}\\ v_{k}^{\theta } \end{array}\right]. \end{array}$$

where it can be observed that although the AP-APBM needs memory from past state estimates $\left[{\hat{x}}_{k-1},\ldots ,{\hat{x}}_{k-l}\right]$, its computational cost is much lower than the cost of the AG-APBM since the dimension of the state is reduced significantly.

## 3. Numerical Simulations

In this section, we implement the proposed AG- and AP-APBM approaches in the context of the high-order Markov models, particularly with the aim of performing state estimation in two common applications: the autoregressive (AR) model problem and the time-delayed control problem.

### 3.1. AR Model

The AR model is one of the most common high-order Markov models. Here, we implemented the proposed approaches to filter the states generated by an AR(3) model based on noisy measurements. Considering the 2-dimensional system state $x={\left[x_{1},x_{2}\right]}^{⊤}$ and the 2-dimensional measurement $y={\left[y_{1},y_{2}\right]}^{⊤}$, the transition and measurement models are given by

(15) $$\begin{array}{cc} x_{k} & =F_{1}x_{k-1}+F_{2}x_{k-2}+F_{3}x_{k-3}+w_{k-1}^{x} \end{array}$$

(16) $$\begin{array}{cc} y_{k} & =Hx_{k}+v_{k}^{y}\;, \end{array}$$

where $w_{k-1}^{x}∼N\left(0,Q^{x}\right)$ and $v_{k}^{y}∼N\left(0,R^{y}\right)$ are additive Gaussian noise with $Q^{x}=\sigma_{x}^{2}I_{2\times 2}$, $R^{y}=\sigma_{y}^{2}I_{2\times 2}$ and $\sigma_{x}=0.1$, $\sigma_{y}=0.1$. $H=I_{2\times 2}$ and

(17) $$\begin{array}{c} F_{1}=\left[\begin{array}{cc} 0.5 & -0.3\\ 0.4 & -0.2 \end{array}\right]\;F_{2}=\left[\begin{array}{cc} 0.2 & 0.1\\ 0.1 & 0.2 \end{array}\right]\;F_{3}=\left[\begin{array}{cc} -0.1 & 0.05\\ -0.05 & 0.1 \end{array}\right]. \end{array}$$

We then compared the performance of the estimation of the filtering process when using the true model above, the PBM-only model, AG- and AP-APBM in the 1st order and 3nd order. It is noted that the true model is constructed based on the AGS method as shown in Equation (7). In the experiments, the PBM refers to the below AR(1) model

(18) $$\begin{array}{cc} x_{k} & =F_{1}x_{k-1}+w_{k-1}^{x}. \end{array}$$

**Figure 1:**
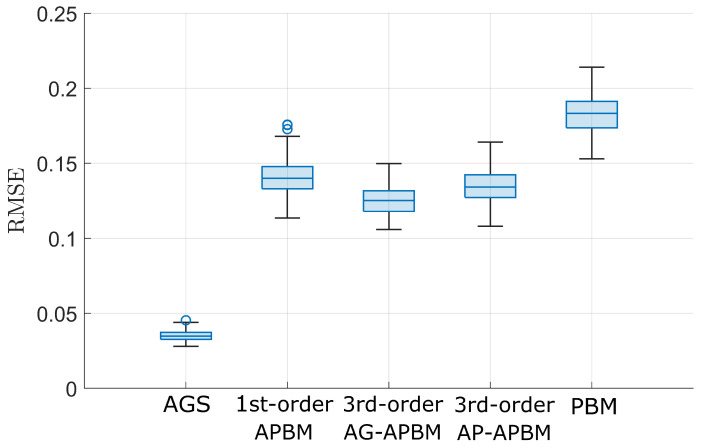
Box plots for the root mean square error (RMSE) of state estimation for the autoregressive-3 (AR(3)) model computed over 200 Monte-Carlo (MC) simulations. The central line in the box indicates the median. The bottom and top edges of the box denote the 25th and 75th percentiles, and the circles denotes the outliers. The AGS and PBM represent the estimation based on the true model and AR(1) model.

**Figure 2:**
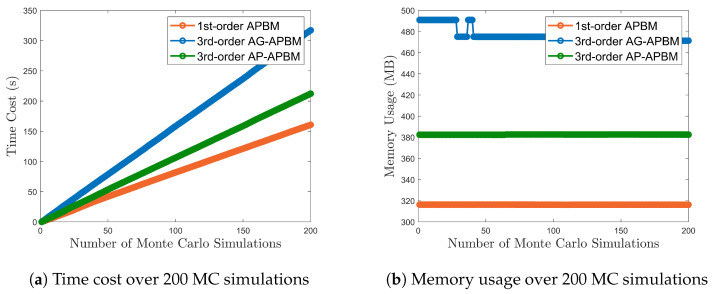
Computational cost over 200 MC simulations. Each MC repetition includes a filtering process of 600 time steps. The simulations were implemented on MATLAB. The cost of AGS and PBM model are not plotted here because the deficiency of training process makes their computational cost incomparable to the hybrid NN models.

The APBM is a linear combination between the PBM and multilayer perceptrons (MLP) as shown below

(19) $$\begin{array}{c} g_{\theta }\left(x_{k-1},\cdot \right)=w_{0}F_{1}x_{k-1}+w_{1}\gamma_{\phi }\left(x_{k-1},\cdot \right), \end{array}$$

where $\theta =[w_{0},w_{1},\phi ]$ and the MLP consists of 1 hidden layer with 5 units and ReLu activation function and output layer with two output units and linear activation function. The second argument in the function (19) can be null for 1st-order APBM, $\left[x_{k-2},x_{k-3}\right]$ for 3rd-order AG-APBM, and $\left[{\hat{x}}_{k-2},{\hat{x}}_{k-3}\right]$ for 3rd-order AP-APBM. Figure 1 shows the root mean square errors (RMSEs) of the estimation using the measurements from the AR(3) model, which are computed based on 200 Monte Carlo (MC) simulations. The PBM gives the largest error because it employs limited knowledge with an AR(1) model, while the true model always achieves the best precision since it has full knowledge of the dynamics model. All three APBMs have better estimation precision compared to the PBM. In terms of the estimate error median, the 1st-order APBM reduces the error from PBM by 22.2%, the 3rd-order AG-APBM reduces the error by 31.1%, and the 3rd-order AP-APBM reduces the error by 26.7%. Among the hybrid learning approaches, the 3rd-order AG-APBM has the best performance since it learns the dynamics through the augmented state with the last two steps. Instead of incorporating the last two steps into the state, the 3rd-order AP-APBM leverages the point estimate of the last two steps as the input of the NN. The median of the RMSE increases about 6.5% compared to 3rd-order AG-APBM, but still lower than the 1st-order APBM. More importantly, the computational cost is significantly reduced by the approximation. Figure 2 shows that the time cost of the 3rd-order AP-APBM decreases by about 37.5% compared with the 3rd-order AG-APBM and the memory usage decreases by about 20% over 200 MC simulations. The simulations are implemented based on the following:

- **Processor (CPU)**: Intel Core i7-10700KF, 8 cores, 3.80 GHz. The multi-core CPU allowed for efficient parallel processing during data preprocessing.
- **Memory (RAM)**: 32 GB DDR4.
- **Storage**: 1 TB NVMe SSD.
- **Operating System**: Windows 11 Pro.
- **Software Platform**: MATLAB R2023a.

It is shown that for AP-APBM, the time cost and memory usage are larger than the cost of the 1st-order APBM, since the extra memory is used to store the approximation $\left\{{\hat{x}}_{k-2},{\hat{x}}_{k-3}\right\}$ when estimating ${\hat{x}}_{k}$. However, the computational cost of the AP-APBM has been greatly reduced compared to the AG-APBM excluding the extra augmented state.

### 3.2. A Delayed-Feedback Control Nonlinear Model

We test the different approaches based on a target tracking problem, where the target is moving on a two-dimensional horizontal plane. The target is operates with an internal feedback control loop, unobserved to the tracker, where communication delays usually occur inside [30,31]. The corresponding dynamics and measurement models are also common in target tracking problems [32]. Considering the state ${\left[x^{⊤},\Omega \right]}^{⊤}$, where $x={\left[p_{x},v_{x},p_{y},v_{y}\right]}^{⊤}$ is the two-dimensional position and velocity, Ω is the turning rate of the target and the feedback control input u, the dynamics model is given by

(20) $$\begin{array}{cc} x_{k} & =Fx_{k-1}+Bu_{k-1}+w_{k-1}^{x}, \end{array}$$

(21) $$\begin{array}{cc} \Omega_{k} & =\Omega_{k-1}+w_{k-1}^{\Omega }, \end{array}$$

where $w_{k-1}^{x}∼N\left(0,\mathrm{diag}(0.1,0.1,0.1,0.1)\right)$, $w_{k-1}^{\Omega }∼N\left(0,10^{-4}\right)$, F defines the constant-velocity model

(22) $$\begin{array}{c} F=\left[\begin{array}{cccc} 1 & T_{s} & 0 & 0\\ 0 & 1 & 0 & 0\\ 0 & 0 & 1 & T_{s}\\ 0 & 0 & 0 & 1 \end{array}\right], \end{array}$$

$T_{s}=1s$ denotes the sampling period, $B=I_{4\times 4}$ is the identity matrix, and $u_{k-1}=s\left(k_{c}G_{k-1}\left(x_{k-3}-{\bar{x}}_{k-1}\right)\right)$ is the input controlled by the difference between the previous state $x_{k-3}$ and reference ${\bar{x}}_{k-1}$. $s\left(A\right):R^{4\times 4}\to R^{4\times 4}$ denotes a saturation function which restricts all elements in the matrix A within [−5,5] due to the possible limitation of the actuator and $k_{c}=1$ is the controller gain. Specifically, we introduce $G_{k-1}$, controlling the state only by the difference between the true velocity and the reference.

(23) $$\begin{array}{c} G_{k-1}=\left[\begin{array}{cccc} 0 & \frac{\sin\Omega_{k-1}T_{s}}{\Omega_{k-1}} & 0 & -\frac{1-\cos\Omega_{k-1}T_{s}}{\Omega_{k-1}}\\ 0 & \cos\Omega_{k-1}T_{s} & 0 & -\sin\Omega_{k-1}T_{s}\\ 0 & \frac{1-\cos\Omega_{k-1}T_{s}}{\Omega_{k-1}} & 0 & \frac{\sin\Omega_{k-1}T_{s}}{\Omega_{k-1}}\\ 0 & \sin\Omega_{k-1}T_{s} & 0 & \cos\Omega_{k-1}T_{s} \end{array}\right]. \end{array}$$

The error is usually defined between the current state $x_{k-1}$ and the reference state ${\bar{x}}_{k-1}$ at the same moment k−1. However, in this case, it is computed based on the state before two steps $x_{k-3}$, due to communication delay, which means the aircraft can access its state until two steps from the current time. (Note that the true state is known to the aircraft but it is unknown to the tracker and is thus estimated by the tracker.) Notice that $G_{k-1}$ introduces the high-order Markovian property into the dynamics. The measurement model is given by the received signal strength and bearings from two collocated sensors

(24) $$y_{k}=\left(\begin{array}{c} 10\log_{10}\left(\frac{\Psi_{0}}{\|p_{0}-p_{k}{\|}^{q}}\right)\\ ∠(p_{0},p_{k}) \end{array}\right)+v_{k}^{y}\;,$$

with $p_{0}={\left(0,0\right)}^{⊤}$ being the position of the sensors, $p_{k}={\left(p_{x,k},p_{y,k}\right)}^{⊤}$ the unknown position of the target, $10\log_{10}\left(\Psi_{0}\right)$=30 dBm, q=2.2 the path loss exponent, $∠(p_{0},p_{k})$ denoting the angle between locations $p_{0}$ and $p_{k}$ in radians, and $v_{k}^{y}∼N\left(0,\mathrm{diag}(1,0.1)\right)$ the measurement noise. The above model can be rewritten as a third-order Markov form

(25) $$\begin{array}{cc} x_{k} & =f\left(x_{k-1},\ldots ,x_{k-3}\right)+w_{k-1} \end{array}$$

(26) $$\begin{array}{cc} y_{k} & =h\left(x_{k}\right)+v_{k}\;. \end{array}$$

### 3.3. AG-APBM and AP-APBM Performance

We implement CKF to estimate the state of interest $x={\left[p_{x},v_{x},p_{y},v_{y}\right]}^{⊤}$ in 200 MC experiments. We will compare the filtering performance of the AGS approach as described in (7), 1st-order Markov APBM in (3), AG-APBM in (10), AP-APBM in (14), pure NN, and pure PBM. It is noted that the AGS approach is considered as the benchmark in this tracking problem, since it has the full knowledge of the dynamics, including the Markovity order and the control input, while the other approaches are not aware of these facts. The constant-velocity model $\bar{f}\left(x_{k-1}\right)=Fx_{k-1}$ is used as the PBM. Here, the APBM is similar to Equation (19), which consists of the PBM and MLP, denoted $\gamma_{\phi }\left(\cdot \right)$ and parameterized by ϕ, with appropriate inputs such that $g_{\theta }\left(x_{k-1},\cdot \right)=w_{0}Fx_{k-1}+w_{1}\gamma_{\phi }\left(x_{k-1},\cdot \right)$, where $\theta =[w_{0},w_{1},\phi ]$. All NNs have one hidden layer with five hidden units and ReLu activation functions, and output layer with dimension $d_{x}=4$ and linear activation functions. It is noted that the six approaches leverage different-level information of the transition model. The AGS has full knowledge of the system, while 1st-order Markov APBM is only given the assumed PBM. The AG-APBM and AP-APBM are aware of the existence of the high-order Markov process, but not the exact order, where they use l=5 instead. Moreover, our reference is designed for the control problem, but the APBM approaches do not have the knowledge of the existence of the reference.

**Figure 3:**
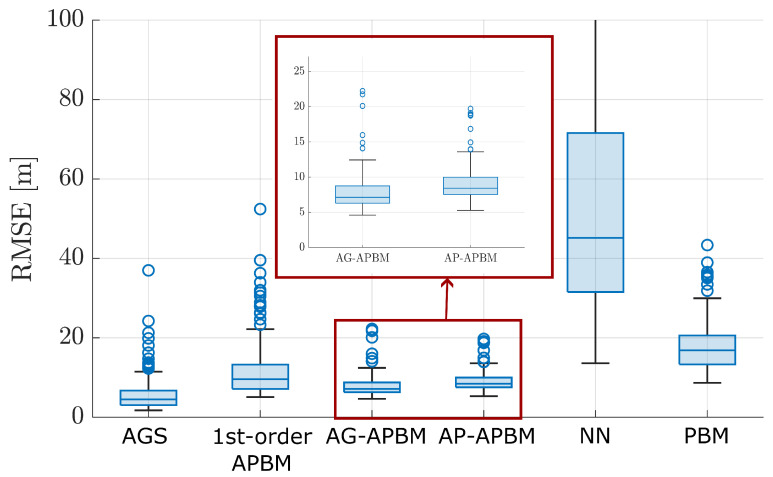
Box plots for the RMSE of position estimations computed over 200 MC simulations. The central line in the box indicates the median. The bottom and top edges of the box denote the 25th and 75th percentiles, and the circles denotes the outliers. The AGS and PBM represents the true model and the constant-velocity model.

**Figure 4:**
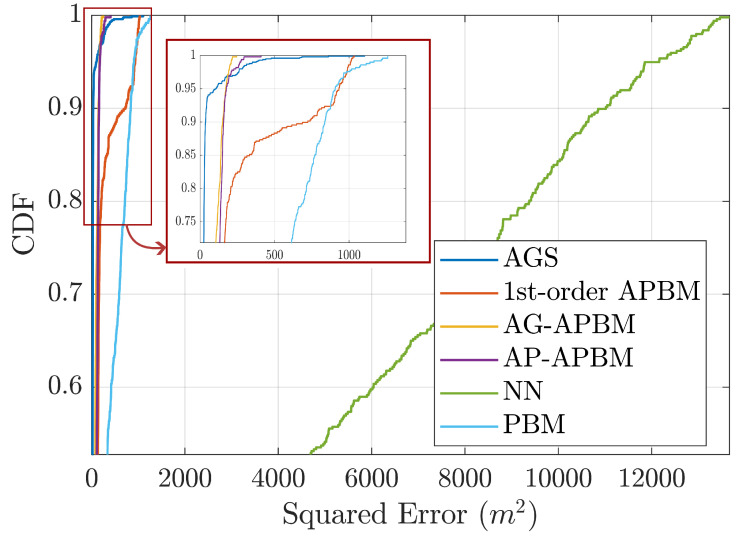
Each curve represents the average empirical cumulative distribution function (CDF) of squared error of position estimation. The average was computed over 200 MC simulations.

Figure 3 shows the box plot of the RMSE of each approach. Figure 4 shows the averaged experimental cumulative distribution function (CDF) over the 200 experiments. Both plots show statistically significant improvements of APBM approaches when compared with both PBM and NNs. In terms of the median, the error of 1st-order APBM is reduced by 41.2%, the error of AG-APBM is reduced by 58.8%, and the error of AP-APBM is reduced by 52.9% compared to PBM. The performances of APBMs are only surpassed by the true model, AGS. When comparing AG- and AP-APBM results the difference is minimal with AP-APBM leading to slightly higher errors. We also highlight that the two proposed approaches led to RMSE values that are very close to the ones obtained with the AGS, despite the perfect knowledge of the order of the Markov process and the transition model assumed in the AGS implementation.

## 4. Conclusions and Future Work

In this study, we extended the APBM framework to deal with high-order nonlinear Markov processes. To this end, we proposed two different implementations with different levels of computational complexity. In the first, we proposed the AG-APBM, where we augmented the state space with past states, thus considerably increasing the number of computations required for the estimation process. In the second approach, AP-APBM, we mitigated the additional complexity by approximating past states by their point estimates, eliminating the state augmentation requirements. Simulated experiments demonstrated the performance of the proposed models in an AR(3) model state estimation and a nonlinear third-order Markov target tracking scenario. The estimation results from the AR model indicated that the high-order AG- and AP-APBM reduced the error by about 30% compared the PBM. Both of them outperformed the legacy 1st-order APBM since they leveraged more state information from the previous steps. Furthermore, the performance of AP-APBM only degrades slightly from the performance of AG-APBM, while significantly reducing the computational cost with 37.5% reduction in time cost and 20% reduction in memory usage based on our simulation environment. The tracking results showed that the AG-APBM and AP-APBM reduced the estimate error by 58.8% and 52.9% compared with the error of PBM even without precise knowledge of the order of the underlying Markov process. Both of them result in a lower RMSE than the standard 1st-order APBM does.

As we mentioned earlier, this article focuses on the state estimation under unknown dynamics (especially the unknown order of Markovity) by learning the system dynamics with PBM. In relation to this, future works could focus on: (1) dealing with unknown noise parameters. The noise statistics is also an important factor in the dynamics system. The noise can be estimated by the maximum likelihood method [29] or the correlation measurement difference method [33]. It is also shown that the data-driven algorithm is effective when learning the noise statistics, e.g., Long short-term memory Kalman filters [34], EKFNet [35] and KalmanNet [36]; (2) considering more general state-space distributions beyond the widely used Gaussian assumption. That is, while APBMs focused on Gaussian models, the general concept can be applied to non-Gaussian setups; and (3) using a different NN structure to learn the high-Markov dynamics. We implemented an MLP to learn the system dynamics in the simulation. The recurrent neural network (RNN), long-short term memory (LSTM) [37], and Transformer [38] may be used due to their mechanisms of forgetting and memorizing the past information especially when the order of the Markovity is not given.

## Data Availability

The original contributions presented in the study are included in the article, further inquiries can be directed to the corresponding author.

## Abbreviations

The article provides a definition for all the symbols and variables that are used in the derivations. For the convenience of the reader, a list of these notations is provided here.

$x_{k}$	State vector at time instance *k*
$y_{k}$	Measurement vector at time instance *k*
$w_{k}^{x}$	Noise of the dynamics model
$v_{k}^{y}$	Noise of the measurement model
h(·)	Possibly nonlinear measurement model
f(·)	Possibly nonlinear true dynamics model
$\bar{f}\left(\cdot \right)$	Physics-based model (PBM)
$\overset{˘}{g}\left(\cdot \right)$	Augmented physics-based model (APBM)
θ	Neural network (NN) parameters
$w_{k}^{\theta }$	Noise of NN parameter dynamics model
$\bar{\theta }$	Pseudo-measurement for NN parameter regularization
$v_{k}^{\theta }$	Noise of NN parameter pseudo-measurement model
${\tilde{x}}_{k}$	Augmented state vector at time instance *k*
$\tilde{g}\left(\cdot \right)$	Augmented-state APBM (AG-APBM)
${\tilde{w}}_{k}^{x}$	Noise of AG-APBM
${\hat{x}}_{k}$	Estimated state at time instance *k*
δ(·)	Dirac delta function
